# Life calendaring as a qualitative strategy to examine human security among queer emerging adult men: a pilot study

**DOI:** 10.3389/fsoc.2024.1322032

**Published:** 2024-01-16

**Authors:** Jerome Visperas Cleofas

**Affiliations:** ^1^Department of Sociology and Behavioral Sciences, De La Salle University, Manila, Philippines; ^2^Doctor of Social Development Program, College of Social Work and Community Development, University of the Philippines Diliman, Quezon City, Philippines

**Keywords:** emerging adulthood, male, qualitative research, research methodology, sexuality

## Abstract

Emerging adulthood has been characterized as a developmental period of insecurities and instabilities, especially among sexual minorities (i.e., queer people). This brief report proposes the utility of life calendaring as a tool to examine how queer emerging adults make sense of their security. First, this paper reviews the basic principles of human security as an approach to human development among emerging adults and explains how sexuality influences their sense of security in their present and projected lives. Second, this report explains the methodological features of life-calendaring as a qualitative research strategy and describes the process of an ongoing life-calendaring-aided interview research project that examines human security among queer emerging adult men. Finally, this article presents key insights from three life calendaring exemplars to demonstrate queering human security in emerging adulthood.

## 1 Introduction: queering human security in emerging adulthood

Emerging adulthood, the transitional phase between adolescence and full-fledged adulthood, has been described as a period of inherent insecurity. During this stage of the life course, individuals grapple with numerous uncertainties and profound changes (Schwartz and Petrova, 2019). With the weight of newfound responsibilities, such as career choices and financial independence, coupled with the desire for self-discovery, emerging adults often find themselves in a constant sense of being “in-between,” oscillating between exhilaration and trepidation (Arnett et al., 2014). They confront daunting questions about their future, interpersonal relationships, and personal values, leading to a pervasive sense of vulnerability and unease as they strive to carve their own paths in an ever-changing world (Arnett et al., 2014; Schwartz and Petrova, 2019). These challenges are accentuated among people from socially disadvantaged backgrounds, including gender and sexual minorities. Given the instabilities and insecurities in this life stage, the theoretical affordance of the human security approach can be used as a lens to understand and address well-being during emerging adulthood.

The United Nations Trust Fund for Human Security (UNTFHS, 2016) describes Human Security Approach (HSA) as a framework that focuses on security at the level of the individual and prioritizes their well-being, freedom, and dignity. This approach recognizes that security extends beyond traditional notions of national security, and encompasses multiple dimensions, including economic, sociocultural, political, physical/health, environmental, and personal security. HSA advocates that individuals must be free from fear, want, and indignity for the achievement of their full human potential. HSA examines and addresses threats and challenges that impact these freedoms while promoting human rights and inclusivity.

Considering the multifaceted challenges, insecurities, and vulnerabilities faced during emerging adulthood (Schwartz and Petrova, 2019), it can be argued that the human security approach is a viable lens to understand the forces that shape well-being during this transitional phase. Beyond the traditional markers of well-being, such as education, employment, and health, HSA also acknowledges the significance of psychosocial factors, such as self-identity, autonomy, and social networks (Schneider, 2018), which are salient during emerging adulthood. Hence, assessing positive development during this stage can be gleaned based on the sense of security that emerging adults have regarding their present and future lives, and the presence/absence of threats to their security. A human security approach to emerging adulthood remains to be unexplored in the literature.

Certain social identities can increase the threats to human security in the time of emerging adulthood, one of which is sexuality. Persons who identify within the queer spectrum of sexuality are more likely to experience enacted and institutionalized stigma (Wittgens et al., 2022). In terms of freedom from fear, queer persons are vulnerable to personal and bodily threats, such as gender-based violence, sexually transmitted infections, and poor access to gender and sexuality affirming healthcare (Stotzer, 2014; Mekler, 2018; Chow et al., 2019). As regards freedom from want, sexual minorities are faced with economic and environmental insecurities, such as discrimination from work, exclusion from social welfare, and lack of gender-sensitive disaster response (Mekler, 2018; Badgett et al., 2019; Goldsmith et al., 2022). As for freedom to live with dignity, queer persons experience political insecurity, due to lack of policies that protect their rights and legislations that punish and disenfranchise sexual minorities (Mekler, 2018; Flores, 2019). However, there is still paucity of evidence that specifically look at HSA in the queer emerging adult population.

Particularly in the Philippines, Christianity remains to be a strong cultural force that reproduces cisheteropatriarchy that maintains the structural violence that threatens the human security of queer Filipinos (Manalastas and Torre, 2013), as evidenced by increased vulnerability to psychological issues (e.g., Cleofas et al., 2022). Queer emerging adults may experience a lack of fair access to work, education, and health care, fear of rejection from family and other social groups, and increased vulnerability to mental health concerns (Salvatore and Daftary-Kapur, 2020). Their experiences as queer persons in the time of emerging adulthood may influence the way they view the security of their future lives.

Building upon earlier research on queering HSA (see Mekler, 2018), more evidence is needed regarding human security in the context of the developmental features of emerging adulthood and how sexual minoritization intersects with the affordances and insecurities found in this life stage. This article proposes the qualitative strategy of life calendaring as a method to examine health security in the time of emerging adulthood.

## 2 Life calendaring, sense of security, and emerging adulthood

As an indicator of well-being, human security can be assessed through objective, quantitative measures (i.e., economic indicators and psychometric scales) and qualitative strategies, by exploring the subjectivities, experiences, and dispositions of individuals and groups (Djuric, 2009). To provide a full picture of the protections and threats to the essential freedoms present in particular social locations, the investigator must assess how individuals make sense of security: how they define a secured life, how they assess the current security of their life, and what personal, social, and environmental forces shape the security of their life.

In line with this, I propose the qualitative method, life calendaring, a strategy that aims to understand how individuals make sense of their present and projected lives to describe psychosocial and well-being phenomena (i.e., human security). Life calendaring includes both their description and evaluation of their present life, and their perspectives, predictions, and perceived possibilities about their future, based on current experiences and contexts. Life calendaring involves the use of a visual temporal aid (e.g., calendar, timetables) paired with a common oral qualitative technique, such as interviews and focus group discussions (FGDs).

Life calendaring combines two visual qualitative strategies that draw reflections from life perspectives. The first is the life history calendar (LHC), which is a tool that facilitates the collection of retrospective data about live events in one or more categories. The popularity of LHC can be attributed to the ability of calendar tools (self-accomplished or researcher-assisted) to increase the accuracy of participants’ memory recall (Glasner and van der Vaart, 2009). However, LHC is limited to retrospective data and past-to-present life events. The second is the future life map (FLM), which is a method that allows the participant to think prospectively about their lives, explore their goals and the possibilities that are ahead of them, and reflect on how their present experiences shape their future orientation (Whitehead and Alves, 2022). However, current applications of FLM deal largely with career planning for young people, and not with overall security or well-being.

Life calendaring integrates the affordances of both research methods to describe and reflect on the present life (LHC), and explore and draw insights from how their aspirations and perceived possibilities in the different developmental stages of their future (FLM). The interviews and/or FGDs among participants are guided by temporal visual cues, such as life pathways and timelines divided into two: present and projected lives. Directed by the researcher and the research phenomenon of interest, the participants are invited to complete the calendars of their lives, indicating the events and conditions existing in their current developmental stage, attempting to envision what might happen in each future life stage, both optimistic and pessimistic outcomes and reflecting on the different factors that shaped their experienced and predicted life trajectories.

By exploring and visualizing present and projected lives as the focal inquiry, the phenomenon of human security is illuminated. The examination of essential freedoms of HSA is not only limited to the positive and negative experiences of well-being among individuals but also should include the optimistic and/or pessimistic future orientation (Djuric, 2009; UNTFHS, 2016). Life calendaring can reveal one’s sense of security, personal agency, autonomy, and control over one’s life circumstances. Moreover, using life calendaring is arguably an appropriate HSA research method for emerging adults as this stage in the life course is one that is characterized by instabilities and possibilities (Arnett et al., 2014; Schwartz and Petrova, 2019). The sense of security and well-being that emerging adults experience during their current stage influences the trajectory and security of the rest of their adulthood (Arnett et al., 2014). To demonstrate its utility for HSA research, the next section features the procedure and insights from a life calendaring study among Filipino queer emerging adult men.

## 3 Life calendaring exemplar: the case of three Filipino queer emerging adult men

Cognizant of the human security issues specific to the developmental stage of emerging adulthood and the additional vulnerabilities experienced by individuals with minoritized sexualities, I embarked on a research that aims to explore the sense of security of the present and projected lives of Filipino queer emerging adult men. This report presents initial insights based on three participants recruited in the early phase of the project.

### 3.1 Life calendaring study procedure

This qualitative research recruited participants with the following inclusion criteria: (1) self-reported gay, bisexual, or queer cis men; (2) aged 18 to 29; (3) has access to Internet connection and videoconferencing applications. The decision to select only cisgender sexual minority men, stems from my (i.e., the researcher) positionality as a cis gay man. Informed consent was secured from informants prior to the scheduling of the interview. I recruited the participants via private online invitations and referrals from social networks. Rapport-building and thorough explanation of the study details helped in retaining participation. The interviews were conducted via Zoom videoconferencing, lasting around 2 h each. The first part of the session was purely an interview guided by the following open-ended questions: (1) *how do you define a “secure life?”*; (2) *how would you describe the security of your current life as an emerging adult?*; (3) *how do you think your being gay/bisexual/queer shapes the security of your life?*

**Figure 1:**
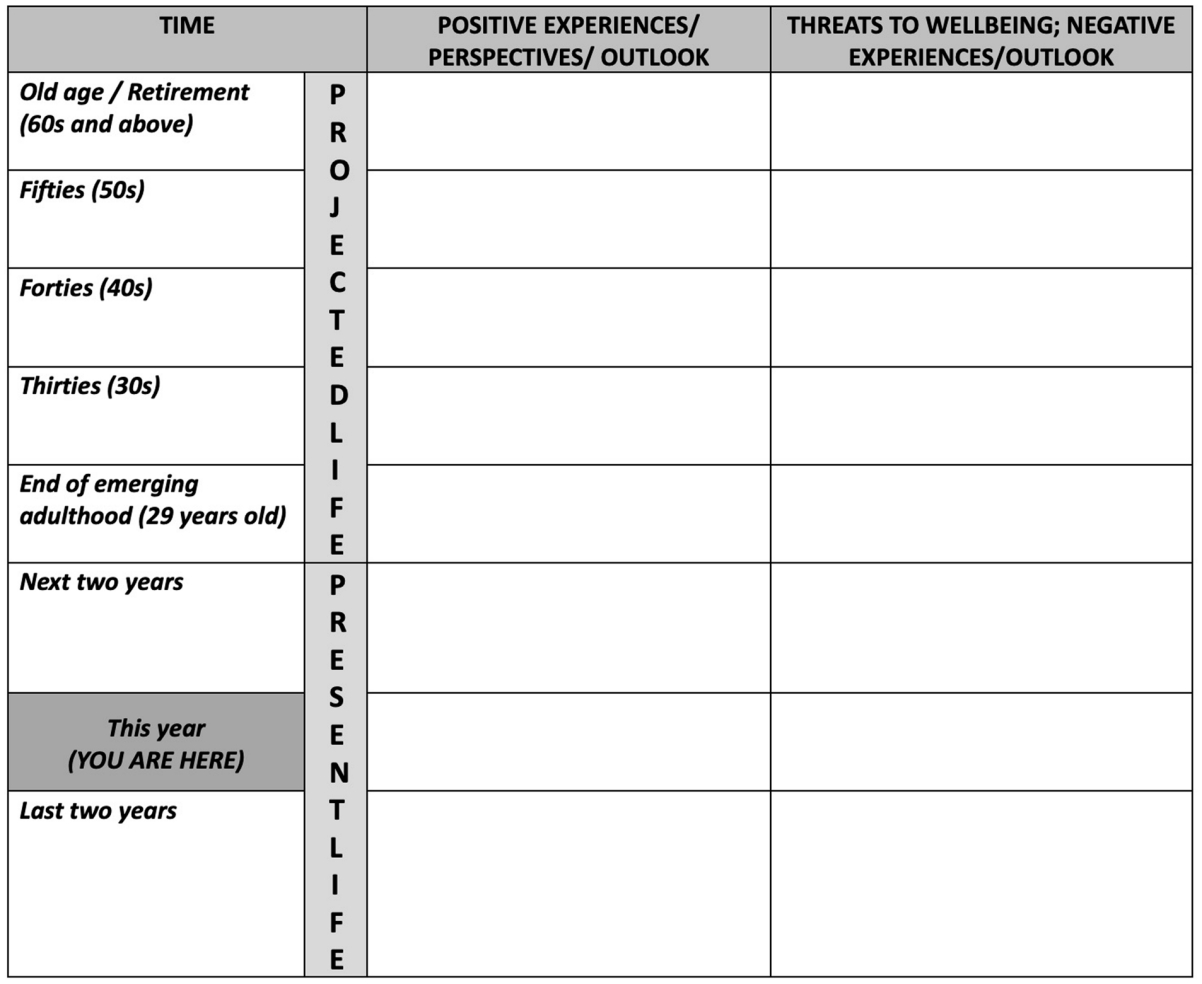
The life calendar visual aid in word processor.

In the second part of the session, the participants were asked to complete a life calendar indicating experiences, events, circumstances, and other factors that are positive (i.e., contributing to security and well-being) and negative (i.e., threats to security and well-being). They were asked to describe their present life, which is defined as the period of 5 years from 2 years prior to the current year, and 2 years after the current year (within the stage of emerging adulthood) based on Faas et al. (2020). Then they were asked to forecast possible outcomes and circumstances in their future, both optimistic and pessimistic. They made predictions for every succeeding decade of their lives (30s, 40s, 50s, 60s and above). As they described their present and projected their future, the Word file of the life calendar (a tabular format with life stages in the rows, and a column for positive and negative life appraisals; see Figure 1) was flashed.

**Figure 2:**
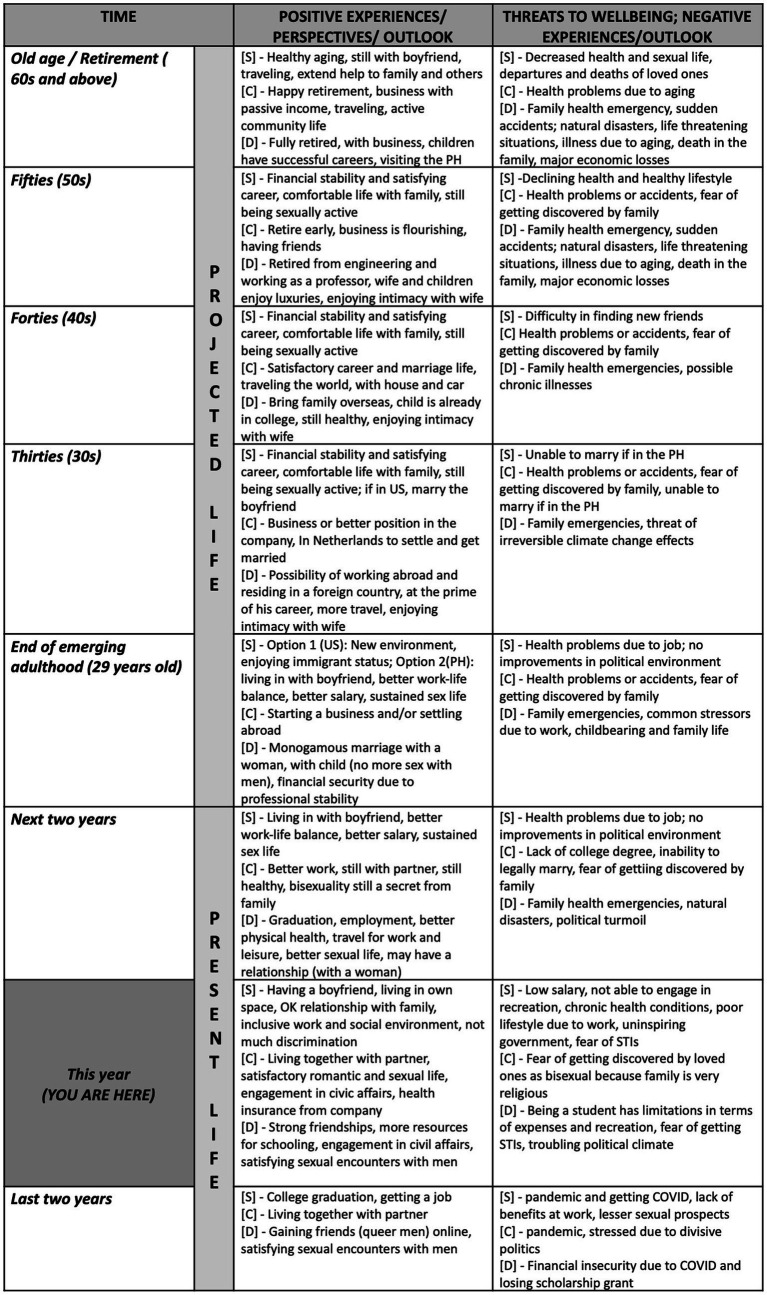
Combined life calendars of Socrates [S], Carlos [C] and Dudley [D].

The visual aid guided the participants on the different life points they were to reflect on. I filled each cell with keywords and phrases that represented their answers for each stage and confirmed its correctness with the participants. After they accomplished the life calendar, I asked them to reflect on whether their constructed life calendar reflected their idea of a “secure life” and how their current emerging adulthood experiences plus their sexuality influenced the way they mapped their imagined future and foreseen threats. After the session, the accomplished life calendar file was sent to the participants, together with a PhP 500.00 worth of food stipend (1USD=PhP56). The studies involving humans were approved by St. Paul University Manila Research Ethics Committee (no. 2023-030-EXT). The studies were conducted in accordance with the local legislation and institutional requirements. The participants provided written informed consent for participation in the study and for the publication of potentially/indirectly identifying information. Pseudonyms were used to label the participants. The next section describes the insights from the life calendaring sessions of three participants in the study. The summarized version of their accomplished life calendars can be visualized in Figure 2.

### 3.2 Socrates (26 years old, gay): two possible locations

Socrates is a data analyst who is in an open relationship with another man during the time of the interview. He mentions, “*for me, a secured life is… happy, with no problems… where I am comfortable to be free and trust the people around [me]…*” He also adds that security during the time of emerging adulthood is being able to take more responsibilities, organize one’s lifestyle, and pay the bills (instead of being dependent on parents). For him, being able to act on his sexual desires freely is important; this is why he agreed to have an open relationship with his partner. These characteristics of security are apparent in the positive experiences and perspectives he indicated in the present section of his life calendar, which includes, having a partner, being with a family who accepts his sexuality, being in an inclusive workplace environment, and having a “*decent number of hook-ups*.” However, his present life also has precarities such as financial and time constraints for recreation, experiencing bodily pains due to work demands, fear of sexually transmitted infections (STIs), and the uninspiring political climate of the country. On the other hand, his forecasted future reveals two pathways. First is migrating to the United States. His projected challenges when he migrates to the US are struggles related to restarting a career path, maintaining a long-distance relationship, and difficulty in finding peer networks. The second pathway is growing old in the Philippines. If he stays, he foresees that his work conditions might give him more physical issues, and if political conditions remain unchanged, he will not be able to legally marry his partner. However, regardless of his eventual residence, he is hopeful that he would have a secured middle adulthood, one that has financial stability, continued intimacy with his partner and other men they desire, and a comfortable life with his family of orientation. Although Socrates foresees sexual decline during old age, he hopes to have a healthy aging, for him to still be able to be intimate with his boyfriend, enjoy traveling, and extend help to his family.

### 3.3 Carlo (22 years old, bisexual): concealment as security

Carlo is a high school graduate who is in a relationship with another man for 4 years. He currently works in the customer service industry. At present, he considers his life to be secure in general. For him, security is being able to live with his partner in one house, having a job that offers health insurance, having enough financial resources for basic needs and recreational traveling. However, he also admits some challenges like being stressed due to the divisive political climate of the country, being in a residence that is prone to flooding, the recent COVID-19 pandemic, and not having a college degree. But his main concern is concealing his sexuality from his very religious family, who currently lives in another town. He says, “*I feel secured [as of now] living away from my family… because my family is obedient to the bible… we have pastors and bishops in the family… I am worried that I’ll be kicked out…*” So, for Carlo in particular, security also means ensuring that his family remains unaware about his desires and current relationship. As for his forecasted future, he imagines a secure middle-aged life with him owning and running his own business, having a good house and a car, engaging in civic organizations for animal welfare, and being able to travel the world. He also mentions, “*yes… I do not see a good life here in the Philippines.*” He imagines his later life in Netherlands, where he could legally marry his partner, and also be away from his religious family. Albeit the physical decline he expects in old age, he foresees a happy and productive retirement, where he receives continuous passive income from his business, living in a satisfying marriage, spending time with friends, engages actively in community life, and travels the world. For him, the threat of being discovered as bisexual by the family will linger for the rest of his life. To keep himself secured, he plans to keep his sexuality a secret because being disowned will affect him negatively.

### 3.4 Dudley (20 years old, bisexual): gay desires today, heterosexual aspirations tomorrow

Dudley is a single man (since birth). He is a college student enrolled in an engineering program. He describes security as, “…*being safe on whatever you do [sic]… it simply means, you are not worried about anything*…” He also mentions aspects of security, like financial security (having enough resources to get by), emotional security (having a stable support system), and “*self security*” (loving oneself). Security during emerging adulthood means being able to have more responsibility and autonomy, and having financial independence. He admits that as a student, he has still not achieved his idea of security. Nevertheless, Dudley’s constructed life calendar shows positive circumstances during his present life. For instance, he reported that he gained strong friendships, had enough resources for schooling, was relatively healthy, and was able to do civic engagements. He also reports having a satisfactory sexual life with men he met on dating apps. However, at the stage of emerging adulthood, he cites challenges such as the COVID-19 pandemic causing health and financial issues for their family, the worsening political turmoil in the country, the risk of contracting STIs, and lack of health insurance. Although Dudley is happy about being sexually active with men at his present life, he plans to settle down and have a heterosexual relationship with a woman, before his emerging adulthood phase ends. When he decides to marry his wife, he will no longer be sexually engaged with men. Hence, a secured middle-aged life for him is having a heterosexual nuclear family of his own, with a good relationship with his wife and children. He also imagines a future where he is in the prime of his career (possibly abroad with his family) and able to travel internationally. However, he also predicts some challenges he may face in the future, like health emergencies and accidents, stresses that are common to work and family life, and possible effects of climate change. While he expects health decline due to aging, he imagines a secured old-aged life as one where he is retired in his profession and now a professor in a university, his children have successful careers and he has grand children, and visiting the Philippines from time to time.

### 3.5 Discussion

This article proposed and described life calendaring as a qualitative strategy to examine human security and other well-being constructs among emerging adults. The utility of life calendaring was demonstrated through a research exemplar that aimed to draw insights from three Filipino queer emerging adult men. The objective was to examine how the case participants made sense of security of their present and projected lives, and how their sexuality influenced their current experiences and imagined futures.

#### 3.5.1 Meanings of a “secured life”

Through the life calendars of the participants, their meanings of security have been revealed more vividly. There are common aspects that characterized human security in their current and projected lives. First is economic, as seen in their aspirations to have successful careers and/or entrepreneurial ventures and achieve financial stability. This notion of security has been identified as an inherent milestone in emerging adulthood (Arnett et al., 2014) and the freedom from want dimension of HAS (UNTFHS, 2016). Second is sexual/romantic, which can be gleaned from their desire to engage in sexual activities and establish intimate relationships with a partner, regardless of gender. Third is recreational, as seen in their desire to travel and enjoy luxuries. Intimacy and leisure-seeking are characteristic desires during emerging adulthood (Arnett et al., 2014), and is a reflection of the freedom to live with dignity (Schneider, 2018). The other aspects can also be characterized in terms of threats to their well-being. For instance, they noted the importance of being healthy and free from illness as requisites to their sense of security. All of them cited the detrimental political environment as negative aspect of their lives, which has also been seen in other developing countries (Mekler, 2018; Badgett et al., 2019; Flores, 2019). Related to this, all three participants have pondered on a future away from the Philippines, where they feel they could experience a more secure life, which is indicative of Manalastas and Torre’s (2013) writings on the lack of social protection for queer Filipinos in their country. These insights corroborate with HSA principles on the essential freedoms and aspects of security to ensure the well-being of individuals (UNTFHS, 2016; Schneider, 2018).

#### 3.5.2 Sexuality and divergences in imagined life trajectories

In relation to the second aim of the study, qualitative insights demonstrate the diversity of life pathways based on the sexuality and sexuality-related experience of individuals. For instance, Socrates projected a future with his family because the latter accepts his sexual identity, while Carlo’s idea of a secure life is concealing his sexuality from his religious family to avoid being disowned; these instances run parallel with the work of Woodford et al. (2015), which indicate the importance of social support for queer emerging adults’ well-being. On the other hand, while Socrates and Carlo see their future as having a relationship with the same sex, Dudley, despite his current homosexual activity, sees a secure future life in a heterosexual union and found a family of his own. These divergent sexual life trajectories confirm Katz-Wise (2015) who noted the sexual fluidity and exploration as a feature of young and emerging adulthood. This confirms prior evidence on how gender and sexuality intersect with HSA (Salvatore and Daftary-Kapur, 2020), particularly on the additional pressures that queer people face in order to achieve over-all well-being and sense of security, as compared to their heterosexual counterparts.

#### 3.5.3 Limitations

Life calendaring as an approach has limitations. It shares the common method bias of LHC and FLM, such as recall bias, lack of contextual detail, subjectivities in interpretation, and lack of standardization. Hence, life calendaring must be paired with other methods (i.e., interviews, FGDs, document analysis) to triangulate the findings to increase their trustworthiness. Lastly, it must be noted that the findings reported in this article are preliminary, given the small sample size and the early stage of the research project, as the goal of this paper is merely to demonstrate life calendaring as a method.

#### 3.5.4 Conclusion

Life calendaring is an appropriate qualitative tool for research endeavors that can gain from examining and visualizing how participants appraise their present and projected lives. Through this method, this brief report demonstrated that queer emerging adults regard economic, sexual, recreational, familial, and political aspects of life as contributory of human security, and how their varying experiences related to sexuality influence their conceived life trajectories.

## Data availability statement

The datasets presented in this article are not readily available because the data are interview narratives, which may potentially expose the identity of participants. Requests to access the datasets should be directed to jerome.cleofas@dlsu.edu.ph.

## Ethics statement

The studies involving humans were approved by the St. Paul University Manila Research Ethics Committee. The studies were conducted in accordance with the local legislation and institutional requirements. The participants provided written informed consent for participation in the study and for the publication of potentially/indirectly identifying information.

## Author contributions

JC: Conceptualization, Data curation, Formal analysis, Methodology, Project administration, Writing – original draft, Writing – review & editing.

## References

[ref1] Arnett J. J. Žukauskienė R. Sugimura K. (2014). The new life stage of emerging adulthood at ages 18-29 years: implications for mental health. Lancet Psychiatry 1, 569–576. doi: 10.1016/S2215-0366(14)00080-7 26361316

[ref2] Badgett M. V. L. Waaldijk K. van der Rodgers Y. M. (2019). The relationship between LGBT inclusion and economic development: macro-level evidence. World Dev. 120, 1–14. doi: 10.1016/j.worlddev.2019.03.011

[ref3] Chow E. P. F. Grulich A. E. Fairley C. K. (2019). Epidemiology and prevention of sexually transmitted infections in men who have sex with men at risk of HIV. Lancet. HIV 6, e396–e405. doi: 10.1016/s2352-3018(19)30043-8 31006612

[ref4] Cleofas J. V. Dayrit J. C. S. Albao B. T. (2022). Problematic versus reflective use: types of social media use as determinants of mental health among young Filipino undergraduates. Health Promot. Persp. 12, 85–91. doi: 10.34172/hpp.2022.11, PMID: 35854847 PMC9277285

[ref5] Djuric S. (2009). Qualitative approach to the research into the parameters of human security in the community. Policing 32, 541–559. doi: 10.1108/13639510910981653

[ref6] Faas C. McFall J. Peer J. W. Schmolesky M. T. Chalk H. M. Hermann A.. (2020). Emerging adulthood MoA/IDEA-8 scale characteristics from multiple institutions. Emerg. Adulthood 8, 259–269. doi: 10.1177/2167696818811192

[ref7] Flores A. R. (2019). *Social acceptance of LGBT people in 174 countries: 1981 to 2017*. Available at: https://escholarship.org/uc/item/5qs218xd

[ref8] Glasner T. van der Vaart W. (2009). Applications of calendar instruments in social surveys: a review. Qual. Quant. 43, 333–349. doi: 10.1007/s11135-007-9129-8, PMID: 20046840 PMC2798968

[ref9] Goldsmith L. Raditz V. Méndez M. (2022). Queer and present danger: understanding the disparate impacts of disasters on LGBTQ+ communities. Disasters 46, 946–973. doi: 10.1111/disa.12509, PMID: 34498778

[ref10] Katz-Wise S. L. (2015). Sexual fluidity in young adult women and men: associations with sexual orientation and sexual identity development. Psychol. Sex. 6, 189–208. doi: 10.1080/19419899.2013.876445

[ref11] Manalastas E. J. D. Torre B. A. (2013). Social Psychological Aspects of Advocating LGBT Human Rights in the Philippines. Gender and Justice Action Research Program, Institute of Human Rights, University of the Philippines Law Center. https://books.google.com.ph/books/about/Social_Psycho-logical_Aspects_of_Advocati.html?id=GsUjMvyH54cC&redir_esc=y

[ref12] Mekler A. G. (2018). “LGBTIQ (in) visibility: a human security approach to SOGIESC” in Routledge handbook of queer development studies. (New York, NY: Routledge), 155–168.

[ref13] Salvatore C. Daftary-Kapur T. (2020). The influence of emerging adulthood on the risky and dangerous behaviors of LGBT populations. Soc. Sci. 9:228. doi: 10.3390/socsci9120228

[ref14] Schneider S. (2018). Tall Tales of danger and security: how a critical human security approach can address major contradictions revealed through a critical narrative analysis of dominant US security strategies. San Francisco, CA: The University of San Francisco.

[ref15] Schwartz S. J. Petrova M. (2019). Prevention science in emerging adulthood: a field coming of age. Prev. Sci. 20, 305–309. doi: 10.1007/s11121-019-0975-0, PMID: 30637671

[ref16] Stotzer R. L. (2014). “Bias crimes based on sexual orientation and gender identity: global prevalence, impacts, and causes” in Handbook of LGBT communities, crime, and justice (New York: Springer), 45–64.

[ref17] UNTFHS. (2016). Human security handbook: an integrated approach for the realization of the sustainable development goals and the priority areas of the international community and the United Nations system. United Nations. Available at: https://www.un.org/humansecurity/wp-content/uploads/2017/10/h2.pdf

[ref18] Whitehead A. Alves N. J. (2022). Use of the “future life map” exercise to improve awareness of career options and opportunities in underrepresented minority undergraduate students pursuing STEM careers. PLoS One 17:e0263848. doi: 10.1371/journal.pone.0263848, PMID: 35143578 PMC8830657

[ref19] Wittgens C. Fischer M. M. Buspavanich P. Theobald S. Schweizer K. Trautmann S. (2022). Mental health in people with minority sexual orientations: a meta-analysis of population-based studies. Acta Psychiatr. Scand. 145, 357–372. doi: 10.1111/acps.13405, PMID: 35090051

[ref20] Woodford M. R. Paceley M. S. Kulick A. Hong J. S. (2015). The LGBQ social climate matters: policies, protests, and placards and psychological well-being among LGBQ emerging adults. J. Gay Lesb. Soci. Serv. 27, 116–141. doi: 10.1080/10538720.2015.990334

